# Oral tranexamic acid for lichen planus pigmentosus inversus: A case report of therapeutic success

**DOI:** 10.1016/j.jdcr.2025.07.037

**Published:** 2025-10-08

**Authors:** Starzyk Tory, Zaki Mehraeil, Remé Brittani, Moon Summer

**Affiliations:** a Department of Dermatology, HCA Healthcare/USF Morsani College of Medicine GME, HCA Florida Largo Hospital, Largo, Florida; b Kansas City University College of Osteopathic Medicine, Kansas City, Missouri; c Department of Internal Medicine, Advocate Christ Medical Center, Oak Lawn, Illinois; d Bay Dermatology & Cosmetic Surgery, Tampa, Florida

**Keywords:** lichen planus pigmentosus, lichen planus pigmentosus inversus, tranexamic acid

## Introduction

Lichen planus pigmentosus (LPP) is an uncommon variant of lichen planus typically found in middle-aged patients of color with dark brown or gray patches on sun-exposed areas such as the face, trunk, and upper extremities.^1^ A rarer variant is lichen planus pigmentosus inversus (LPPI), which, in contrast to LPP, more commonly affects Caucasians and has a predilection for intertriginous regions like the axillae, groin, abdominal folds, and antecubital and popliteal areas.^2^ Few case reports describe effective treatments for LPP, and even fewer on LPPI. Topical tretinoin and tacrolimus 0.1% ointment are the most commonly used treatments; however, responses vary.^3^ To our knowledge, this is the first report to demonstrate the detailed use and favorable effects of oral tranexamic acid (TXA) in improving hyperpigmentation in a patient with LPPI.

## Case report

A 60-year-old female presented with hyperpigmented patches in the bilateral axillae. These patches were cosmetically distressing to her and had been present for 1 year. Empirically, she was diagnosed with chromhidrosis and trialed clindamycin phosphate 1% solution twice daily and benzoyl peroxide 10% cleanser daily for 3 months without improvement. At this point, a shave biopsy of the right axillary vault was performed, which demonstrated lichenoid dermatitis. Over the next year, she trialed several topical treatments without improvement, including tacrolimus 0.1% ointment, betamethasone 0.05% cream, and halobetasol/tazarotene 0.01%/0.45% lotion. Topical depigmenting agents were not trialed.

**Fig 1:**
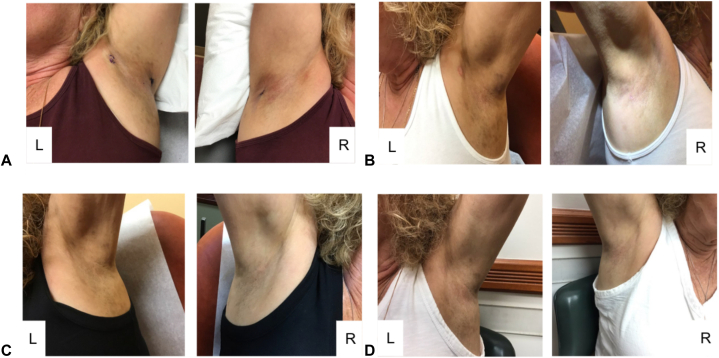
**A,** Initial presentation: hyperpigmented patches in bilateral axilla. Two punch biopsies of the right axillary vault and left posterior axilla were performed, and a shave biopsy of the left anterior axilla was performed. **B,** Month 1: mild improvement after 1 month of TXA 250 mg daily. **C,** Month 6: significant improvement after continuing TXA 250 mg daily for 2 more months and increasing dose to 500 mg daily for 3 months. **D,** Month 9: maintaining improvement after decreasing TXA dose for 3 months to 250 mg daily. *TXA*, Tranexamic acid.

After 1 year of failed topical treatments, her presentation developed to include the bilateral popliteal skin, left cavum concha, right crus of helix, and bilateral axillae (Fig 1, *A*). Two punch biopsies of the right axillary vault and left posterior axilla and a shave biopsy of the left anterior axilla were performed. Histopathology demonstrated subtle vacuolar change with pigment incontinence consistent with LPPI and left posterior axilla gram-positive cocci consistent with impetiginization. The patient denied any contraindications to oral TXA, such as renal, cardiovascular, or pulmonary disease; malignancy; history of thrombolytic disease or bleeding disorder; and hypersensitivity to oral TXA. The patient had no recent surgeries, no longer smokes, and is not on anticoagulant or hormone therapy. Baseline coagulation profile was not performed. Oral TXA 250 mg daily was prescribed through a compounding pharmacy along with topical mupirocin 2% ointment for the impetiginized areas. After 1 month, she noticed decreased hyperpigmentation in the bilateral axillae (Fig 1, *B*). At 3 months, she noted significant improvement, and TXA dosage was increased to 500 mg daily. At 6 months, the patient had marked resolution in hyperpigmentation, was satisfied with the treatment, and felt more confident wearing sleeveless shirts (Fig 1, *C*), and TXA was decreased back to 250 mg daily. At 9 months, the patient maintained improvement (Fig 1, *D*), and her dosage was titrated down to 250 mg every other day for 2 weeks, then every third day for 2 weeks, then every fourth day for 4 weeks, then every fifth day for 4 weeks, and finally discontinued. At the 12-month follow-up, patches remained stable, and tacrolimus 0.1% topical ointment was prescribed daily for maintenance. Throughout treatment with TXA, the patient denied side effects.

## Discussion

LPPI has an unclear etiology, but some associations have been linked to hepatitis C and tight clothing.^2^^,^^4^ Our patient’s husband had a history of hepatitis C infection; however, she tested negative. She works with hair dyes daily, but no other identifiable triggers were noted. LPPI has very few cases documented in medical literature and even fewer case reports on treatment options. Most commonly, topicals like tretinoin and tacrolimus 0.1% ointment are used, but results vary and patches may be recalcitrant to topical steroids and hydroquinone.^3^^,^^5^

Some case reports showed that oral TXA in LPP was helpful, with many patients experiencing complete resolution of their pruritus and complete disappearance of their lesions.^6^, ^7^, ^8^ Only 1 case report briefly highlighted the use of oral TXA in LPPI; however, no results were shared.^9^ The proposed mechanism of action of oral TXA for LPPI is that by reducing plasmin-mediated prostaglandin and arachidonic acid, there is an anti-inflammatory effect that reduces CD8+ T-cell activity and keratinocyte apoptosis.^5^^,^^9^ Decreased damage to basal keratinocytes reduces melanin dropping and improves hyperpigmentation.^10^

Given the paucity of information on treatment regimens for TXA in LPPI, our dosage regimen was guided based on the patient’s response to treatment. By slowly titrating and tapering the dose, the patient avoided adverse effects and had sustained results. Significant improvement in pigmentation was observed with each follow-up visit, showing a notable reduction in the intensity and extent of hyperpigmentation. In our case, the patient was started on 250 mg oral TXA daily for 3 months, and a dramatic improvement was noticed. Then, the dosage was increased to 500 mg daily for 3 months, and at 6 months, the patient’s postinflammatory hyperpigmentation had resolved. Then, the dosage was decreased to 250 mg daily for the following 3 months to find a maintenance dose. At 9 months, the dosage was further titrated down. At the 12-month follow-up, the patches remained stable, and TXA was discontinued. The use of TXA in LPPI is promising, as seen in this case. Given the paucity of cases reported using this treatment in LPP and LPPI, further studies are needed with a larger sample size to establish dosing, duration of treatment, maintenance, and long-term outcomes. This case supports adding TXA to the list of treatment options for LPPI, especially in patients who do not respond to topical treatments.

## Conclusion

This case report is the first to highlight the efficacy of oral TXA as a treatment option for LPPI, a challenging hyperpigmentary disorder with limited treatment options. Our patient had significant improvement in hyperpigmentation while using oral TXA and maintained results through the 1-year follow-up visit. This case supports incorporating oral TXA in the treatment arsenal for LPPI. However, further research with more extensive clinical studies is required to determine ideal oral TXA dosing regimens, duration, and maintenance in treating LPPI.

## Conflicts of interest

None disclosed.
